# Factors of time and genetics influencing the rate of monozygotic twinning following ART

**DOI:** 10.5935/1518-0557.20250016

**Published:** 2025

**Authors:** Adriana Bos-Mikich, Gabriella M. Andrade, Nilo Frantz

**Affiliations:** 1 Universidade Federal do Rio Grande do Sul, Porto Alegre, RS, Brazil; 2 Nilo Frantz Reproductive Medicine, Porto Alegre, RS, Brazil

**Keywords:** Assisted reproduction, gemelarity etiology, monozygotic gestations, twins

## Abstract

**Objective:**

The incidence of monozygotic twinning (MZT) increases following ART procedures. Despite numerous studies, no definitive conclusion has been reached regarding the potential etiology of MZT after fertility treatments. This study aims to analyze the possible factors influencing the incidence of MZT gestations over an 8-year period.

**Methods:**

This retrospective cohort study analyzed 21 autologous (patient´s own oocytes) and heterologous (donor oocytes used) ICSI cycles involving single or double embryo transfers that resulted in monozygotic gestations between 2015 and 2022 at a single IVF center. The frequency and type of monozygotic gestation were recorded and analyzed on a yearly basis.

**Results:**

We observed a significant clustering of MZT gestations in two years (2018 and 2019) during the study period. Additionally, two cases of heterologous MZT gestation occurred following egg donation from the same donor across two different oocyte collection cycles.

**Conclusions:**

Our observations suggest that not a single factor related to ART procedures causes MZT after fertility treatment. Maternal genetic and epigenetic factors, as well as uncontrolled environmental factors, may work synergistically to promote the occurrence of monozygotic twinning.

## INTRODUCTION

For several decades, multiple births resulting from transferring more than one embryo to the uterus were a major concern associated with infertility treatments using assisted reproduction technologies (ART). Advances in embryo culture conditions (Gardner & Lane, 1997) allowed for the selection of a single optimal embryo for transfer, significantly reducing multiple gestations without affecting birth rates. Nonetheless, another form of multiple pregnancy persists with increased frequency following ART treatments: monozygotic twinning (MZT).

Monozygotic (identical twin) pregnancies can occur spontaneously in humans through natural conception at an estimated low frequency of 0.4% (MacGillivray, 1986). Edwards *et al*. (1986) were the first to highlight a higher-than-normal rate of MZT following IVF treatment. The authors suggested that the conditions of *in vitro* fertilization and embryonic growth might predispose individuals to MZT. However, Derom *et al*. (1987) soon challenged this assumption by demonstrating that the artificial induction of ovulation alone for intrauterine insemination, without oocyte collection, in vitro fertilization, or embryo culture, was the mechanism responsible for the increased MZT frequencies.

From those early reports to the present day, several studies and opinions have been put forward to explain the association between the increased occurrences of MZT after ART, but without a conclusive response. The variety of putative factors present during IVF treatment cycles that may be involved in monozygotic gemelarity ranges from ovarian stimulation with exogenous hormones to culture conditions and embryo manipulation, such as zona pellucida breach for ICSI and genetic testing. Additionally, a survey among IVF patients with multiple gestations in their families revealed a hereditary component to the observed MZT rates after ART (Sobek *et al*., 2015).

Despite the extensive literature on the origins of MZT after ART, only one report has described the “clustering” of MZT gestations during specific periods. Here, we report a similar clustering phenomenon observed while analyzing monozygotic gestation rates from 8 years of IVF records at a private clinic.

## MATERIAL AND METHODS

We reviewed records of ART cycles that resulted in MZT gestations at the Nilo Frantz Reproductive Medicine Center. The frequency and type of MZT pregnancies achieved through the use of autologous or heterologous oocytes in embryo transfer cycles were analyzed from 2015 to 2022. The study was conducted in accordance with the ethical standards set by the institutional research committee, and all patients had previously given informed consent for the use of their data. MZT pregnancy was defined by ultrasound around 5 to 6 weeks of gestation when more than one fetal pole with cardiac activity was identified in a single gestational sac or when the number of fetal poles with cardiac activity exceeded the number of embryos transferred.

Patients underwent ovarian stimulation protocols using either a GnRH agonist or antagonist, along with transvaginal ultrasound examinations starting on treatment days 6 to 8. When at least one follicle reached 14mm or more, either 250µg of recombinant hCG (Ovidrel; EMD Serono) or 10,000 U of urinary hCG (Novarel; Ferring Pharmaceuticals) was administered subcutaneously. Ultrasound-guided oocyte retrieval was performed 36 hours after hCG administration. During the period from 2015 to 2022, embryos were cultured using Continuous Single Culture (CSCM^TM^, FUJIFILM *Irvine* Scientific), and the embryo transfer policy primarily involved frozen blastocyst transfers.

## RESULTS

A total of 21 MZ gestations occurred after embryo transfers between 2015 and 2022. Of all MZ pregnancies, 18 resulted from autologous ART cycles, and three resulted from donor oocyte cycles. Three different cycles of donor oocyte collection yielded a total of 90 oocytes (34 from the first cycle, 45 from the second cycle, and 11 from the third cycle), of which 72 were at MII (33, 29, and 11 MII for the first, second, and third cycles, respectively). The first recipient received 8, the second 10, and the third 11 MII oocytes. The mean age among the 18 autologous MZ pregnancies was 33 years. The ages of the two egg donors were 22 and 23 years. Notably, two of the three heterologous MZ gestations resulted from oocyte donations from the same donor. Except for one case (in 2015), all transfers were frozen blastocyst transfers. Of these, 14 were single blastocyst transfers, one was a cleavage stage transfer, and six involved two blastocysts transferred.

Table 1 depicts the cycle characteristics of autologous and heterologous cycles performed between 2015 and 2022 that resulted in MZ gestations.

**Table 1:** Characteristics of cycles that resulted in autologous and heterologous MZ gestations.

	Autologous (n=18)	Heterologous (n=3)
Patient age (mean year)	33.0	42.0
Oocytes collected (n)	270	90
Matured oocytes (n)	217	73
Fertilization rate (mean %)	81.7	85.3
Blastocyst rate (mean %)	59.9	69.2
Single embryo transfer (n)	15	3
No. of embryos transferred (n)	24	3
PGT performed (n)	6	2
Cleavage stage transfers (n)	1	0
Blastocyst stage transfers (n)	17	3

Table 2 describes the type of gemelarity observed after single and double embryo transfers in autologous and heterologous IVF cycles. The most frequent form of gemelarity after a single embryo transfer was dichorionic twinning. Among the monochorionic gemelarities, it was not possible to distinguish between monoamniotic and diamniotic gestations due to a lack of records on fetal membranes. Two cases of single embryo transfers resulted in the birth of six babies, specifically one monochorionic gestation in an autologous cycle and one trichorionic gestation in a heterologous cycle. One double blastocyst transfer in an autologous cycle resulted in a trichorionic gestation with three babies born. All nine babies from these three transfers were boys. An additional case of monochorionic twin boys was detected after the transfer of two embryos, while the second gestation resulted in a baby girl. On the other hand, among the dichorionic gestations conceived after the transfer of a single embryo, the majority of babies born were girls (n=9 out of 13 births). In one trichorionic gestation after the transfer of two blastocysts and in one chorionic gestation after the transfer of one blastocyst, only one baby was born. Overall, there were 32 babies born, comprising 20 boys and 12 girls, nearly twice as many boys as girls. No malformations were detected in any of the newborns.

**Table 2:** Characteristics of autologous and heterologous MZ gestations after single or double embryo transfer.

	1 ET												2 ET								
**AT**	1 GS (n=3)^*^				2 GS (n=9)^**^				3 GS (n=0)				1 GS (n=2)			2 GS (n=1)			3 GS (n=3)		
	HB	BB	Sex ♀♂		HB	BB	Sex ♀♂		HB	BB	Sex ♀♂		HB	BB	Sex ♀♂	HB	BB	Sex ♀♂	HB	BB	Sex ♀♂
	7	7	0	7	18	11	7	4	-	-	-	-	4	2	2 0	3	3	1 2	9	4	0 4
**HT**	1 GS (n=0)				2 GS (n=2)				3 GS (n=1)												
	HB	BB	Sex ♀♂		HB	BB	Sex		HB	BB	Sex ♀♂										
	-	-	-	-	4	2	2	0	3	3	0	3									

Legend: ET - embryo transferred; GS - gestational sac; HB - heartbeat; BB - baby born; Sex - baby sex; AT - autologous cycles; HT - heterologous cycles.^*^ 2 x 2 fetuses/GS and 1 x 3 fetuses/GS.^**^4 x 2GS /0 BB; 5 x 2 GS/2BB; 1 x 2 GS/1BB.

Another noteworthy observation is that two heterologous gestations resulted from embryos from oocytes obtained from the same donor during two different stimulation cycles nearly one year apart. One patient had three gestational surrogacies, resulting in the birth of three boys, while the second recipient had two gestational surrogacies that ended in abortion, with no baby born.

**Figure 1:**
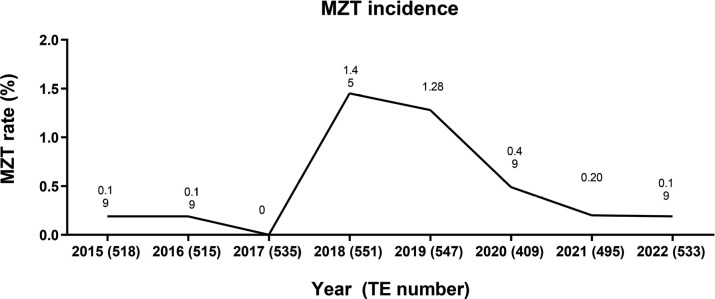
Monozygotic twinning (MZT) incidence along the study period.

The frequency of MZT distribution along the eight-year interval showed remarkable clustering in 2018 and 2019, when the incidence of MZ gemelarity reached values seven to eight times higher than in previous years and three to four times more elevated than in the subsequent years of 2020 and 2022 (Fig. 1).

## DISCUSSION

The present analysis of assisted reproduction cycles at a single IVF center in southern Brazil highlights the occurrence of temporal clustering of MZ gestations following embryo transfer, as previously reported by Vaughan *et al*. (2016). Similar to the present study, the earlier report was conducted in a single urban fertility center and demonstrated that MZT occurred in clusters during four of 24 six-month intervals. In contrast to the earlier report, our analysis did not detect the same clustering effect in six-month intervals but rather at yearly intervals. These observations lead us to believe that the triggering factor for MZT gestations may be related to an environmental or methodological component from the IVF center, which promotes multi-fetal pregnancies via maternal influences. The putative factors may include a range of possibilities, such as changes in equipment, cleaning products, culture medium suppliers or brands, superovulation drugs, and external factors like unusual outbreaks of viral disease, among many others. Thus, the lack of agreement among previous reports regarding embryonic factors that may be linked to the significant increase in MZT gestations after ART suggests that a combination of maternal and environmental components may predispose the occurrence of multiple pregnancies in IVF cycles.

The embryonic origin of MZT has been challenged by various studies demonstrating that monozygosity does not increase after single blastocyst transfer compared to a single cleavage stage embryo (Papanikolaou *et al*., 2010) or after zona breach for PGD examination (Verpoest *et al*., 2009). The study by Gu *et al*. (2018) indicated that ICM incarceration, potentially caused by zona breaching, is not associated with increased MZT rates. However, a recent study suggested that assisted hatching may elevate the risk of MZT in older women (>37 years old) (Liu *et al*., 2022).

On the other hand, the analysis of MZT across four groups of ART procedures- IVF, ICSI, TESA, and PGT (Li *et al*., 2023)- showed no significant differences in monozygosity among patients undergoing any of these techniques, regardless of whether the transfers were fresh or frozen embryo transfers. These results are similar to data from a large multicentric study (Kadam *et al*., 2023), which analyzed single cleavage and blastocyst transfers. It is important to note that the study by Li *et al*. (2023) found no differences in neonatal outcomes (congenital malformations and birth weight) of monozygotic twins among the four ART groups. Thus, the possibility of a common laboratory or infertility clinic factor does not seem to be involved in the increased occurrence of monozygotic twins after ART treatment, as concluded by Scaravelli *et al*. (2022) following a multicenter retrospective cohort study in Italy.

Together, this data strengthens the notion that it may not be embryo manipulation *per se* and culture that serves as the source of MZT in ART; rather, MZT may have a maternal origin. Blickstein (2005) proposed that the common denominator for zygotic splitting in ART and after ovulation induction lies *in vivo* rather than *in vitro*. According to the author, factors present before fertilization, during follicle growth, and oocyte maturation may cause the zygote to split at an early stage during the initial cleavages due to cell repulsion, as previously suggested by Hall (1996), which goes undetected by embryologists. Additionally, the potential role of ovulation induction and superovulation as sources of MZT (Deron et al., 1987) may be supported by evidence showing a defined spatial location of proteins and regulatory molecules in mouse and human oocytes (Antczak & Van Blerkom, 1997). The precise location of these molecules may stem from surrounding follicle cells through directed secretion during follicle growth and oocyte maturation. Under normal physiological circumstances, the female reproductive tract provides an optimal environment for the developing oocyte and embryo. Ovarian stimulation with exogenous hormones alters the environment of the follicle and oocyte (Hyttel *et al*., 1989; Sirard *et al*., 2006; Krisher, 2013). In line with this evidence, a recent review by Raffaeli & Stern (2020) suggested that early amniotic embryos, including humans, possess cytoplasmic components or nongenetic mechanisms to prevent the occurrence of MZT. Thus, unknown environmental disturbances that occur during artificial ovarian stimulation protocols may alter the distribution or effectiveness of cytoplasmic inhibitory factors, allowing MZT to occur occasionally. Maternal environmental stressors that impact follicle growth and oocyte quality are known to affect oocyte and embryo quality in domestic animals. However, so far, there is no clear evidence of similar environmental stressors affecting human gamete quality and reproduction (Gallo *et al*., 2020).

On the other hand, the putative mechanisms by which ooplasmic components are disrupted, potentially allowing for the occurrence of MZ, may be operating alongside a hereditary factor that increases the likelihood of MZ gestations. The maternal origin of monozygosity is further supported by our observation of two cases of egg donation cycles involving the same donor, who underwent two superovulation cycles and donated her oocytes to two different recipients across different years, both of which resulted in MZ gestations. The two cases of MZ gestations in recipients who received oocytes from the same donor strongly suggest a maternal genetic component influencing the occurrence of MZT, as previously described by Sobek *et al*. (2015).

In addition to the heritable genetic factors, one cannot exclude the epigenetic component affecting MZ gestations, as there appears to be an increased frequency of defects in children born after ART, which are known to be associated with epigenetic and imprinting errors related to IVF techniques (Sciorio & El Hajj, 2022).

Among the twin pregnancies described here, the majority of cases (16/21; 76%) were multichorionic (dichorionic or trichorionic). This observation reinforces the hypothesis of cell repulsion during an early developmental stage as the mechanism behind the occurrence of MZ gestations. According to this hypothesis, blastomeres may have segregated due to an ooplasmic factor to form new independent entities, without any morphological changes detectable by the embryologist. Conversely, the high frequency of multichorionic pregnancies following single embryo transfers challenges the assumed theory of early embryo splitting, as indicated by Sundaram *et al*. (2018).

We encountered three cases of monochorionic multiamniotic pregnancies following the transfer of a single embryo. Only one of these cases underwent PGD. These pregnancies indicate that ICM splitting due to ZP trapping during hatching is not the sole mechanism to explain MZT but may represent an additional factor contributing to the frequency of monochorionic, multiamniotic twin gestations. Other studies have failed to establish a relationship between ZP breaching techniques and MZT (Schachter *et al*., 2001; Milki *et al*., 2003; Blickstein, 2005; Gu *et al*., 2018). Gu *et al.* (2018) showed that fully-hatched blastocysts resulted in an MZT rate of 2.1%, similar to that observed with 8-shaped blastocysts (2.3%). Consistent with this finding, complete removal of the ZP through pronase digestion prior to blastocyst transfer did not prevent monozygotic pregnancies following IVF (Frankfurter *et al*., 2004). Therefore, even if the zona may be involved in certain cases of MZT, it is unlikely to be the exclusive mechanism.

We did not find a high miscarriage rate associated with MZT pregnancies in this study. Only six out of the 21 (28%) MZT pregnancies ended in miscarriage. Thirty-two babies were born, with 20 boys and 12 girls. The results for live births are significantly better than previous reports for MZ gestations (Gu *et al*., 2018; Jones & Benirschke, 1983; Livingston & Poland, 1980). We had two cases of singleton births, indicating the vanishing twin syndrome (one case of trichorionic gestation after the transfer of two blastocysts and one case of dichorionic gestation after transferring one blastocyst). The prevalence of baby boys does not appear to be related to the type of monozygosity, whether monochorionic multiamniotic or multichorionic, and we have no explanation for this sex discrepancy.

## CONCLUSION

A combination of factors, mainly maternal *in vivo* components ranging from genetic and epigenetic to controlled ovarian stimulation and putative/unknown external environment agents occurring in specific periods of time, act synergistically to alter the ooplasm and generate MZT pregnancies after ART treatments. Due to the high risk, both for the mother and for the babies, the chance of an MZT gestation occurring after the transfer of a single embryo should always be discussed with IVF patients.

## References

[r1] Antczak M, Van Blerkom J. (1997). Oocyte influences on early development: the regulatory proteins
leptin and STAT3 are polarized in mouse and human oocytes and differentially
distributed within the cells of the preimplantation stage
embryo. Mol Hum Reprod.

[r2] Blickstein I. (2005). Estimation of iatrogenic monozygotic twinning rate following
assisted reproduction: pitfalls and caveats. Am J Obstet Gynecol.

[r3] Derom C, Vlietinck H, Derom R, Van den Berghe H. (1987). Increased monozygotic twinning rate after ovulation
induction. Lancet.

[r4] Edwards RG, Mettler L, Waiters DE. (1986). Identical twins and in vitro fertilization. J in Vitro Fert Embr Transf.

[r5] Frankfurter D, Trimarchi J, Hackett R, Meng L, Keefe D. (2004). Monozygotic pregnancies from transfers of zona-free
blastocysts. Fertil Steril.

[r6] Gallo A, Boni R, Tosti E. (2020). Gamete quality in a multistressor environment. Environ Int.

[r7] Gardner DK, Lane M. (1997). Culture and selection of viable human blastocysts: a feasible
proposition for human IVF?. Hum Reprod Update.

[r8] Gu YF, Zhou QW, Zhang SP, Lu CF, Gong F, Tan YQ, Lu GX, Lin G. (2018). Inner cell mass incarceration in 8-shaped blastocysts does not
increase monozygotic twinning in preimplantation genetic diagnosis and
screening patients. PLoS One.

[r9] Hall JG. (1996). Twins and twinning. Am J Med Genet.

[r10] Hyttel P, Greve T, Callesen H. (1989). Ultrastructure of oocyte maturation and fertilization in
superovulated cattle. Prog Clin Biol Res.

[r11] Jones KL, Benirschke K. (1983). The developmental pathogenesis of structural defects: the
contribution of monozygotic twins. Semin Perinatol.

[r12] Kadam N, Woodhead G, Kellam L, Campbell A, Jayaprakasan K. (2023). Odds and Predictors of Monozygotic Twinning in a Multicentre
Cohort of 25,794 IVF Cycles. J Clin Med.

[r13] Krisher RL. (2013). In vivo and in vitro environmental effects on mammalian oocyte
quality. Annu Rev Anim Biosci.

[r14] Li Y, Chang Q, Mai Q. (2023). Pregnancy and neonatal outcomes of monozygotic twins resulting
from assisted reproductive technology: a 10-year retrospective
study. Reprod Biol Endocrinol.

[r15] Liu C, Su K, Liu G, Shang W, Wang X, Li C, Chen L, Zhou X. (2022). The Impact of Assisted Hatching on Monozygotic Twinning is
Related to Female Age and Insemination Method: A New
Perspective. Twin Res Hum Genet.

[r16] Livingston JE, Poland BJ. (1980). A study of spontaneously aborted twins. Teratology.

[r17] MacGillivray I. (1986). Epidemiology of twin pregnancy. Semin Perinatol.

[r18] Milki AA, Jun SH, Hinckley MD, Behr B, Giudice LC, Westphal LM. (2003). Incidence of monozygotic twinning with blastocyst transfer
compared to cleavage-stage transfer. Fertil Steril.

[r19] Papanikolaou EG, Fatemi H, Venetis C, Donoso P, Kolibianakis E, Tournaye H, Tarlatzis Devroey P. (2010). Monozygotic twinning is not increased after single blastocyst
transfer compared with single cleavage-stage embryo transfer. Fertil Steril.

[r20] Raffaeli A, Stern CD. (2020). Signaling events regulating embryonic polarity and formation of
the primitive streak in the chick embryo. Curr Top Dev Biol.

[r21] Scaravelli G, Pisaturo V, Levi Setti PE, Ubaldi FM, Livi C, Borini A, Greco E, Villani MT, Coccia ME, Revelli A, Ricci G, Fusi F, Costa M, Migliorati E, De Luca R, Vigiliano V, Bolli S, Reschini M. (2022). Monozygotic twin rate among ART centers: a multicenter analysis
of data from 18 Italian units. J Assist Reprod Genet.

[r22] Schachter M, Raziel A, Friedler S, Strassburger D, Bern O, Ron-El R. (2001). Monozygotic twinning after assisted reproductive techniques: a
phenomenon independent of micromanipulation. Hum Reprod.

[r23] Sciorio R, El Hajj N. (2022). Epigenetic Risks of Medically Assisted
Reproduction. J Clin Med.

[r24] Sirard MA, Richard F, Blondin P, Robert C. (2006). Contribution of the oocyte to embryo quality. Theriogenology.

[r25] Sobek A, Zbořilová B, Procházka M, Šilhánová E, Koutná O, Klásková E, Tkadlec E, Sobek A. (2015). High incidence of monozygotic twinning after assisted
reproduction is related to genetic information, but not to assisted
reproduction itself. Fertil Steril.

[r26] Sundaram V, Ribeiro S, Noel M. (2018). Multi-chorionic pregnancies following single embryo transfer at
the blastocyst stage: a case series and review of the
literature. J Assist Reprod Genet.

[r27] Vaughan DA, Ruthazer R, Penzias AS, Norwitz ER, Sakkas D. (2016). Clustering of monozygotic twinning in IVF. J Assist Reprod Genet.

[r28] Verpoest W, Van Landuyt L, Desmyttere S, Cremers A, Devroye P, Liebaers I. (2009). The incidence of monozygotic twinning following PGD is not
increased. Hum Reprod.

